# Laboratory Features of Newly Diagnosed Multiple Myeloma Patients

**DOI:** 10.7759/cureus.4716

**Published:** 2019-05-22

**Authors:** Azhar Hussain, Hana Farag Almenfi, Abdelfattah M Almehdewi, Mohammed S Hamza, Malpe Surekha Bhat, Narasimha Prasad Vijayashankar

**Affiliations:** 1 Medicine, Xavier University School of Medicine, Oranjestad, ABW; 2 Internal Medicine, Khaled Ben Al-Walid Clinic, Tripoli, LBY; 3 Laboratory Medicine, Higher Institute of Medical Professions, Benghazi, LBY; 4 Nutrition, Faculty of Public Health / Benghazi University, Benghazi, LBY; 5 Biochemistry, Xavier University School of Medicine, Oranjestad, ABW; 6 Pharmacology, Xavier University School of Medicine, Oranjestad, ABW

**Keywords:** multiple myloma, esr, hypercalcemia, nlr, non-secretory myeloma

## Abstract

Objectives: Multiple myeloma (MM) is a malignant disorder characterized by proliferation of a single clone of plasma cells derived from β-cells in the bone marrow. It is the second most common adult hematological malignancy, and it is the most common cancer with skeletal components as its primary site. The purpose of the retrospective study was to assess the hematological profile, different biochemical parameters, and the serum electrophoresis patterns of patients consistent with clinical symptoms of multiple myeloma.

Materials and methods: A retrospective study of 99 patients diagnosed with multiple myeloma (MM) was carried out at the Hematology Department of Benghazi Medical Center (BMC) in Benghazi, Libya from January 2010 to March 2017. Information on the laboratory features was obtained at presentation (before treatment) and analyzed.

Results: Of the 99 study detected cases of multiple myeloma at diagnosis, 14% were younger than 45 years and 35% were 70 years or older. The mean age was 61 years, of which 42 (42.4%) were males and 57 (57.6%) were females. Anemia was seen in roughly half of the diagnosed cases, most of which was normocytic normochromic anemia. High erythrocyte sedimentation rate (ESR) was seen in 65.3% of cases and increased neutrophil-to-lymphocyte ratio (NLR) was seen in 29.7%. Other abnormal serum levels with regard to the cases are as follows: hyperproteinemia in 30%, low albumin/globulin (A/G) ratio in 54.2%, hypercalcemia in 11.3%, serum creatinine level of >2.0 mg/dL in 27.2% cases, and increased β2-microglobulin in 67%. Serum protein electrophoresis revealed a localized band in 70.8% of patients. Monoclonal bands were seen in 44 cases (95.7%) and a bi-clonal pattern in two cases (4.3%), 78% of M-band showed migration to γ-region of electrophoretogram and 18% to β-region.

Hypogammaglobulinemia was detected in 32.8% and hypergammaglobulinemia was detected in 49.2%. Of the hypergammaglobulinemia, 18.1% showed polyclonal gammaglobulinemia. Bence Jones protein was positive in 50% cases. IgG was the commonest type, followed by IgA then light chain. In 26.5% of cases, the only diagnosis was multiple myeloma. Light chain multiple myeloma patients had high α2 globulin concentration and normal A/G ratio. Apart from the diagnosis of multiple myeloma, a number of cases had varying diagnoses including the following: 4% non-secretory myeloma, 2% amyloidosis with nephrotic syndrome, 2% liver cirrhosis, and 18.2% renal failure. Most patients presented in stage III.

Conclusions: The presence of anemia, high ESR, and low A/G ratio in elderly patients should alert the clinician to investigate along the lines of multiple myeloma. In this study, unfortunately, the laboratory investigations were insufficient for diagnosing this disease in most patients. Most patients were diagnosed at stage III. Absence of paraprotein in the blood does not exclude multiple myeloma. It was further observed that most of the patients presented with significant renal damage, which attributed to hyperuricemia, hypercalcemia, or high NLR. Multiple causes of renal failure occur in myeloma and are often present at the time of diagnosis.

## Introduction

Multiple myeloma (plasma cell myeloma, myelomatosis, or Kahler disease) is a rare form of cancer, with approximately 114,000 new cases globally per year. It is the second most common adult hematological malignancy, and the most frequent site to involve is the skeleton [1]. It is characterized by neoplastic proliferation of a single clone of plasma cells derived from B-cells in the bone marrow, which produce an abnormal immunoglobulin called monoclonal protein (M protein) and free light chain proteins designated as kappa or lambda. The M protein is a tumor marker specific for monoclonal gammopathies because it reflects the clonal proliferation of immunoglobulin. The normal immunoglobulin is comprised of the heavy and light chains. In regard to the type of immunoglobulin produced, multiple myeloma (MM) can be classified into IgA MM, IgD MM, IgE MM, IgM MM, IgG MM, light chain (L.C) MM, and non-secretory MM [2]. These malignant plasma cells have the potential to affect many bones in the body, possibly resulting in compression fractures as well as lytic bone lesions. Hypercalcemia, renal impairment, anemia, and bone lesions (CRAB) symptoms are the currently accepted diagnostic criteria for the diagnosis of symptomatic (and therefore treatable) myeloma [3,4]. There is a slight male predominance, the median age at onset is 66 years, and only 2% of patients are younger than 40 years of age at diagnosis [5]. The primary cause of multiple myeloma is idiopathic, but radiation exposure to industrial and agricultural toxins and possible genetic predisposition may be etiological factors in certain cases. Although MM is not curable, treatment with medications is often effective enough to induce a period of remission. A stem cell transplant is an option for some patients [3-5].

Serum protein electrophoresis (SPE) should be undertaken whenever multiple myeloma is suspected. SPE is the mainstay laboratory test for the detection of abnormal monoclonal gammopathy proteins. M protein is generally observed as a localized band, which is frequently seen on gamma or beta regions; it may also be seen on alpha-2-globulin regions in rare situations [6,7]. Very rarely bi-clonal gammopathy can also be seen [8]. The nature of the monoclonal protein is then characterized and confirmed by an immunofixation electrophoresis (IFE). The bone marrow aspiration shows the presence of plasma cells or myeloma cells, varying from less than 1% to over 90%, depending on the degree of involvement in the site of the marrow aspirated. A diagnosis usually relies on the presence of at least 10% of the bone marrow nucleated cells being plasma cells. In Libya, the annual mortality rate per 100,000 people from multiple myeloma was assessed by the Global Burden of Disease (GBD) study in 2013; it has increased by 34.6% since 1990, an average of 1.5% a year. At 18.4 deaths per 100,000 men in 2013, the peak mortality rate for men was higher than that of women, which was 8.2 per 100,000 women [9].

Aim of the study

The objectives of the present study were the following.

1. Identify initial basic laboratory features of multiple myeloma (MM).

2. Assess the biochemical, hematological profile and electrophoretic pattern consistent with clinical symptoms of multiple myeloma.

3. Aid physicians to entertain a high index of suspicion and therefore target their investigations in order to prevent late presentation and avert complications.

4. Correlate the results of these investigations together with their appropriate statistical analysis and interpretation.

## Materials and methods

Subjects

All patients referred to the outpatient clinic of the Hematology Department in Benghazi Medical Center (BMC) from January 2010 to March 2017 were analyzed before treatment. Permission was obtained from the Research Ethics Board in the hospital. Out of the total 104 patients with multiple myeloma, five cases with complete remission state were excluded in the present study. Thus, a total of 99 patients were analyzed. There were 57 females with age ranging from 40 to 80 years and 42 males with age from 38 to 80 years. The diagnosis of multiple myeloma was based on the following findings: (1) increased numbers of abnormal, atypical, or immature plasma cells in the bone marrow or histological proof of plasmacytoma; (2) presence of an M protein in the serum; or (3) bone lesions consistent with those of multiple myeloma.

Method

In this retrospective study, the laboratory data (biochemical, hematological and electrophoretic pattern) obtained at the time of diagnosis was extracted from patients’ medical records. The data were analyzed after anonymization of patients. Poor data filing and documentation was a major setback in this study. For instance, although we assessed 99 patients, records for city distribution were available only in 79 patients, and those for M-spike distribution were available only in 49 patients.

The serum protein fractions analyzed included total protein, albumin, α1-globulin, α2-globulin, β1-globulin, β2-globulin, and γ-globulin. Laboratory parameters evaluated included hemoglobin, leukocytes, thrombocytes, erythrocyte sedimentation rate, plasma cells, serum calcium, uric acid, creatinine, β2 microglobulin and lactate dehydrogenase levels, liver function test (LFT), and serum electrolytes. NLR was calculated using data obtained from the complete blood count (CBC). Bone marrow studies were also included.

Statistical analysis

We used Microsoft Excel version 2007 for data analysis and found the frequency for respective parameters. Demographic data was explored as expressed in figures.

## Results

**Figure 1:**
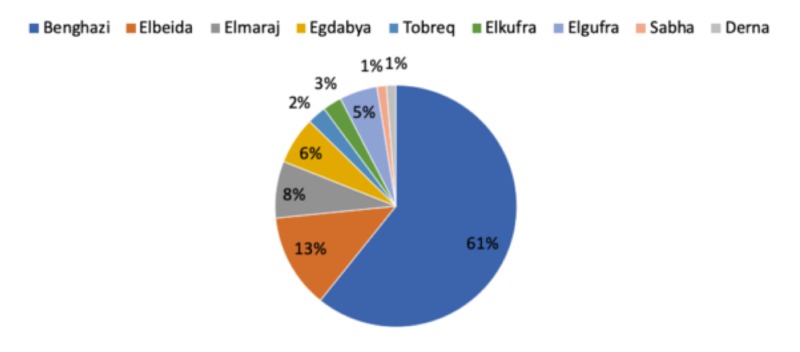
Distribution of patients (n=79) according to the major cities of Libya

The distribution of patients according to the major cities of Libya (n=99) is shown in Figure 1. The mean age of incidence of the assessed 99 patients was 61.8±11.3 years and there was a female prevalence (57 out of 99 cases). In this study, 79.8% of cases were detected in the age group 50-80 years with the peak incidence in the age group 61-70 years (34.34%), and 23.23% > 70 years (Table 1).

**Table 1:** Age distribution of patients

Age Group (Years)	Male	Male %	Female	Female %	Total	Total %
< 40	2	2.02	1	1.01	3	3.04
41-50	5	5.05	12	12.12	17	17.17
51-60	11	11.11	11	11.11	22	22.22
61-70	11	11.11	23	23.23	34	34.34
>70	13	13.13	10	10.10	23	23.23

Anemia (Hb <10 gm/dl) was initially found in 50.5% cases and was mostly normocytic normochromic. Table 2 shows hematological parameters of patients with mean hemoglobin level at 10.2±2.1 gm%. ESR was 74±49; >50 mm/1hr was seen in 65.3% cases, >100 mm/1hr was seen in 27% cases. WBC was 6.89±3.28; leukopenia was found in 19.3%, while leukocytosis was found in 9.1%, NLR (>4) was seen in 29.7%. The mean platelet count was 254±148 with thrombocytopenia in 14.2% and thrombocytosis in 6.1%.

**Table 2:** Hematological profile of patients with multiple myeloma WBC - white blood cell, ESR - erythrocyte sedimentation rate, NLR - neutrophil-to-lymphocyte ratio

Parameter	Mean ± SD
Hemoglobin (gm%)	10.2 ± 2.1
WBCs (10^3^/μL)	6.89 ± 3.28
Platelets (10^3^/μL)	254 ± 148
Plasma cells %	66 ± 3.4
ESR (mm/hr)	74 ± 49
Neutrophils %	64.15 ± 14.4
Lymphocyte %	26.2 ± 12.8
NLR	4.01 ± 3.93

Table 3 shows the biochemical parameters of the patients, with total protein and albumin, mean levels 8.08 ± 2.3 and 3.4 ± 0.75, respectively. Hyperproteinemia (> 9 gm/dl) was seen in 30% and hypoproteinemia (< 6 gm/dl) in 10% cases. A/G ratio was decreased (< 0.80) in 54.2% and high (>2) in 4.4%. In 30% of patients with normal total proteins, A/G ratio was low. Hypercalcemia (>11 mg/dl) was seen in 11.3% and hypocalcemia (<8 mg/dl) in 9% cases with mean level 9.4±1.5. Serum creatinine level > 2.0 mg/dL was seen in 27.2% of cases, the mean was 2.9±2.1. The mean level of LDH was 438±253. β2 microglobulin was raised in 67% with mean level 2.7±0.95. Glutamate aminotransferases were high at 6.3%. Hypernatremia was seen in 5.9%, hyponatremia in 5.9%, hyperkalemia in 10.4% and hyperuricemia in 50% of cases. The mean concentration of individual protein fractions α1, α2, β1, β2, γ-globulin was 4.66±1.88, 10.2±3.7, 6.2±8.2, 9.4±13.5, 23.1±16.7 (gm/dL), respectively (Table 4).

**Table 3:** Biochemical profile of patients with multiple myeloma

Parameter	Mean ± SD
Total protein (gm/dL)	8.08 ± 2.3
Albumin (gm/dL)	3.4 ± 0.75
Calcium (mg/dL)	9.4 ± 1.5
Creatinine (mg/dL)	2.9 ± 2.1
Lactate dehydrogenase (U/L)	438 ± 253
β2-microglobulin (g/dL)(n=25)	2.7 ± 0.95

**Table 4:** Electrophoretic pattern of patients with multiple myeloma

Parameter	Mean ± SD
α1-Globulin (gm/dL)	4.66 ± 1.88
α2-Globulin (gm/dL)	10.2 ± 3.7
βı -Globulin (gm/dL)	8.2 ± 6.2
β2-Globulin (gm/dL)	13.5 ± 9.4
γ-Globulin (gm/dL)	23.1 ± 16.7

**Figure 2:**
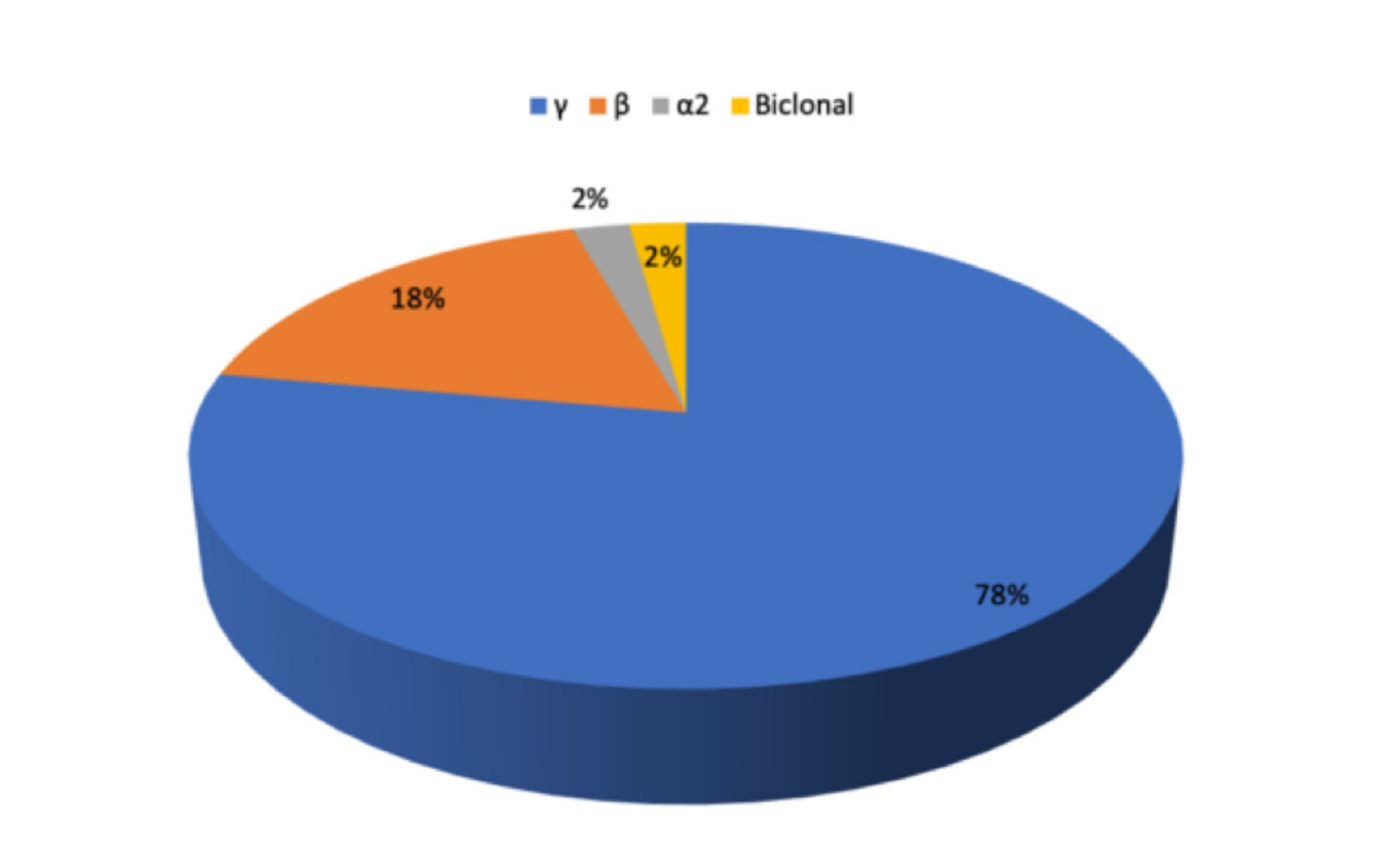
Location of M-spike in serum protein electrophoresis (N=45)

Serum protein electrophoresis revealed a localized band in 70.8% (n=65) of patients, showed monoclonal band in 44 cases (95.7%) and a bi-clonal pattern in two cases (4.3%). We found that 78% of M-band showed migration to γ-region of electrophoretogram, 18% to β and 2% to α2-region as well as bi-clonal regions (Figure 2). Hypogammaglobulinemia was detected in 32.8%, and hypergammaglobulinemia in 49.2%. We also found that 18.1% of hypergammaglobulinemia showed polyclonal gammaglobulinemia. Bence Jones protein was positive in 50% cases. The results revealed that 40.4% of patients had IgG PP type, 18.2% had IgA, 3% had light chain, 2% had IgM, and 1% had IgD. In 26.5% of cases, the individuals were diagnosed as multiple myeloma only (Table 5). Differences in IgG PP detection rate were found between men (41.0%) and women (59.0%). IgA PP and L.C were found to be 66% and 63.6%, respectively, in men, and 34% and 36.4%, respectively, in women.

**Table 5:** Type of monoclonal component L.C - light chain

Type of M protein	Number	Percentage %
MM	26	26.26
IgG	21	21.21
IgG κ	12	12.12
IgG λ	7	7.07
IgA	13	13.13
IgA κ	2	2.02
IgA λ	3	3.03
IgM	1	1.01
IgM κ	1	1.01
IgD λ	1	1.01
L.C	3	3.03
Free κ only	4	4.05
Free λ only	3	3.03
Bi-clonal IgA	1	1.01
Bi-clonal IgG λ	1	1.01

**Figure 3:**
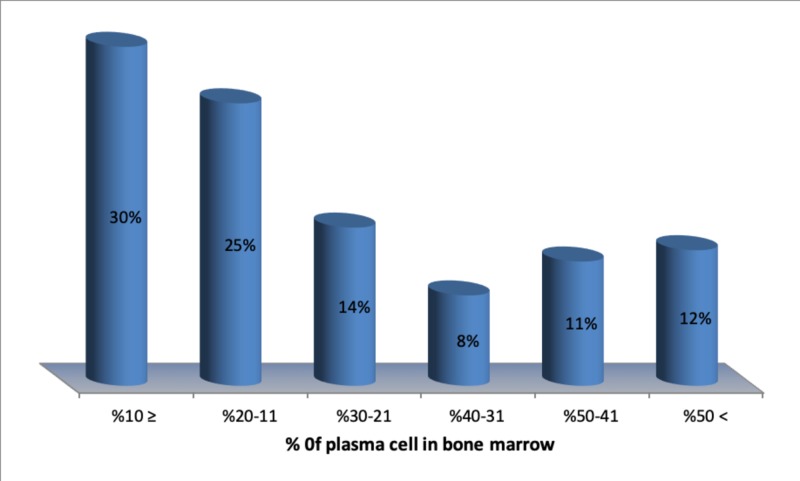
Plasma cell in bone marrow aspirate (n=71)

All L.C MM patients had high α2 globulin concentration and normal A/G ratio; 58.3% of this group had hypoproteinemia, 41.7% were normal, in 80% there was no M-spike. In majority of the cases (70%), the plasma cell was >10%. In 12% of cases, it was more than 50% (Figure 3). Non-secretory myeloma was recognized in 4% of patients, amyloidosis with nephrotic syndrome in 2% (caused by IgA), and liver cirrhosis in 2%. The incidence of renal failure in our study was high (18.2%), 50% of this group showed hyperuricemia, 33% hypercalcemia, 27.8% high NLR (>6) and 16% severe hyperproteinemia. Most patients presented in stage III.

## Discussion

In this study, most of our patients had been diagnosed at stage III of disease, which is indicative of late presentation or aggressive disease. Although poor patient records were the major drawback of this study (total n = 99, records for most parameters were available for less than 99 patients), the authors have analyzed the records within the constraints of what best could be retrieved. Since the numbers for the parameters were large enough for statistical analysis, the authors strongly believe that the poor records should not affect the analysis much. In Libya, there is no routine healthy-adult yearly evaluation, hence patients who present with symptoms, present with more advanced stages of the disease. This may partially explain the poor outcome. The mean age of incidence was 61.8 years and there was a female prevalence whereas Dash [7], Kapoor [10], and Phekoo [11] reported mean age of 64, 65, and 73 years, respectively, and found a male prevalence. Anemia was initially found in 50.5% cases and median hemoglobin concentration was 10.2 gm/dl as compared to other studies [5,11-13]. The anemia was mostly normocytic normochromic. ESR was seen to be elevated in most of the cases (65.3%) and a value of >100 mm/hr was observed in (27%) cases, whereas Diwan reported 100% cases with elevated ESR in his study [14]. Leukopenia was seen in 19.3%, thrombocytopenia in 14.2%, thrombocytosis in 6.1% similar to the study by Diwan [14].

A high neutrophil-to-lymphocyte ratio (NLR) has been reported to have a negative prognostic impact and an independent prognostic factor for overall survival in MM patients [15-17]. In this study, the median NLR was 3.94 (range 0.48-19.5), NLR (>4) was detected in 29.7% of the 10 patients (from 2010 up to 2013) that were followed-up and alive at the time of analysis (2017); there was high NLR in two cases at the initial diagnosis.

Serum albumin is a significant prognostic factor that reflects the severity of disease progression. In the present study, albumin levels were low in about 55% of cases. This is similar to previous studies that observed low albumin levels in MM patients [13,18]. Low A/G ratio was associated with high risk for liver and hematologic malignancies (which includes multiple myeloma) [19]. Reversal of the albumin/globulin ratio was another commonly observed presenting feature in this patient group (54.2%)--these represent simple and affordable diagnostic tools that may further inform the choice of early referral. Majority of the patients did not have a renal impairment at presentation (81.8%). This finding is similar to that reported by other studies [20-22].

Renal function impairment is a common phenomenon in multiple myeloma. In this present study, renal failure was associated with hypercalcemia (33%), hyperuricemia (50%), and severe hyperproteinemia (16%). The most common type of M protein found in MM is IgG followed by IgA and light chain only. Our study showed that IgG accounted for 40.8% of cases and IgA accounted for 18.5% of patients. We found that 70.8% of patients in the study group had the presence of M band on serum protein electrophoresis as against the 100% reported by Diwan [14]. Out of 46 positive cases, M-spike was localized in the gamma region (γ) in 78% cases, beta region (β) 18% cases, and α2 region in 2% cases. Bi-clonal gammopathy was noted in two cases (2%) in contrast to an earlier study [11]. Chopra et al. reported that 84.8% of the cases had an M band in the gamma (γ) region and that 15.2% cases had an M spike in the β- region [23]. Tripathy reported M spike in the γ- region in 87.5% cases and 12.5% cases in the β- region [24], O’Connell noted that M spike in α2 region was IgA [25], whereas in this study, it was ⱪappa L.C. A bi-clonal gammopathy is suspected when there are two proteins with different mobilities comprising two different monoclonal heavy chains with their respective monoclonal light chains [26]. A bi-clonal gammopathy may also consist of two heavy chains of the same class and monoclonal light chains of the same type. In this study, there were two cases of bi-clonal M-band on the same (γ) region: IgG λ and IgA. However, in other studies, it was found to be in different regions [8].

Hypogammaglobulinemia was detected in 32.8%, whereas O’Connell reported only 10%. The mean bone marrow plasma cells were 66%, and >10% in 70% of cases as against the 31% and >10% in 92% cases reported by Dash [14]. In normal individuals, plasma cells constitute 1% in the bone marrow but in multiple myeloma, tumor load in bone marrow increases up to 80% depending on severity [27]. However, some disease features such as amyloidosis with nephrotic syndrome have been found to be quite rare.

In agreement with the findings of an earlier study, there were four cases of non-secretory MM present [5]. Of the four cases, there were two cases in which serum-free light chain assay was not done. Due to this, the introduction of the assays to quantitate circulating free ⱪ and free λ light chains have made possible the reclassification of approximately one half to two-thirds of patients previously thought to have non-secretory myeloma [28]. β2M has been confirmed as a highly significant prognostic factor in each study in which it has been examined; it reflects tumor burden and renal impairment but, in this study, there were only 25 cases measured. All in the L.C MM patients group had high α2 globulin concentration, although, α2 may be relatively higher owing to a coexisting acute phase response (haptoglobin) or to preferential retention of larger molecules (α2-macroglobulin). In 80% of this group (L.C MM) patients had negative M-spike, and all of them had normal or low total protein levels.

To avoid late presentation and avert complications, we recommend consultation with a hematologist when the diagnosis of multiple myeloma is strongly suspected. Diagnostic values of A/G ratio are needed to consolidate the results. Serum protein electrophoresis should be routinely used in all elderly patients with features of bone pain, anemia, high ESR, and hypercalcemia. More importantly, the addition of the serum free light chains (FLC) assay to serum protein electrophoresis (SPEL) and immunofixation electrophoresis (IFE) is recommended in all newly diagnosed patients with plasma cell dyscrasia and it makes the serum diagnostic studies sufficiently sensitive. Since the chain portion of the whole non-IgG immunoglobulin may not be detected in isoelectric focusing (IEF), free light chain assay may be required to rule out misdiagnosis and is essential in patients with the non-secretory disease.

## Conclusions

Multiple myeloma, a cancer originating from plasma cells, characteristically grows in an out-of-control manner and accumulates in the bone marrow throughout the body. Untreated myeloma may directly progress to damage bones resulting in a dangerous elevation in serum calcium levels potentially leading to fractures. Antecedent risk factors, such as hyperproteinemia and hypercalcemia could precipitate cast nephropathy and inflammation resulting in acute kidney injury which may eventually progress to chronic kidney disease (CKD). Myeloma cells interfere with blood production and suppress normal antibody production, resulting in anemia and decreased immunity, leaving the patients vulnerable to infection. In this study, unfortunately, the laboratory investigations were insufficient for diagnosing this disease in most patients prior to stage III. Diagnosed patients presented with renal function complications that could be attributed to hyperuricemia and hypercalcemia. Proper diagnosis at an early stage of multiple myeloma is necessary for preventing related complications and providing sufficient treatment.
